# Enhanced Sensitivity for Detection of HIV-1 p24 Antigen by a Novel Nuclease-Linked Fluorescence Oligonucleotide Assay

**DOI:** 10.1371/journal.pone.0125701

**Published:** 2015-04-27

**Authors:** Peihu Fan, Xiaojun Li, Weiheng Su, Wei Kong, Xianggui Kong, Zhenxin Wang, Youchun Wang, Chunlai Jiang, Feng Gao

**Affiliations:** 1 School of Life Sciences, Jilin University, Changchun, Jilin, China; 2 National Engineering Laboratory for Acquired Immune Deficiency Syndrome Vaccine, Jilin University, Changchun, Jilin, China; 3 Key Laboratory for Molecular Enzymology & Engineering, Jilin University, Changchun, Jilin, China; 4 State Key Laboratory of Luminescence and Applications, Changchun Institute of Optics, Fine Mechanics and Physics, Chinese Academy of Sciences, Changchun, Jilin, China; 5 State Key Laboratory of Electroanalytical Chemistry, Changchun Institute of Applied Chemistry, Chinese Academy of Sciences, Changchun, Jilin, China; 6 Division of Human Immunodeficiency Virus/Acquired Immune Deficiency Syndrome and Sex-transmitted Virus Vaccines, National Institutes for Food and Drug Control, Beijing, China; Institut National de la Santé et de la Recherche Médicale, FRANCE

## Abstract

The relatively high detection limit of the Enzyme-linked immunosorbent assay (ELISA) prevents its application for detection of low concentrations of antigens. To increase the sensitivity for detection of HIV-1 p24 antigen, we developed a highly sensitive nuclease-linked fluorescence oligonucleotide assay (NLFOA). Two major improvements were incorporated in NLFOA to amplify antibody-antigen interaction signals and reduce the signal/noise ratio; a large number of nuclease molecules coupled to the gold nanoparticle/streptavidin complex and fluorescent signals generated from fluorescent-labeled oligonucleotides by the nuclease. The detection limit of p24 by NLFOA was 1 pg/mL, which was 10-fold more sensitive than the conventional ELISA (10 pg/mL). The specificity was 100% and the coefficient of variation (CV) was 7.8% at low p24 concentration (1.5 pg/mL) with various concentrations of spiked p24 in HIV-1 negative sera. Thus, NLFOA is highly sensitive, specific, reproducible and user-friendly. The more sensitive detection of low p24 concentrations in HIV-1-infected individuals by NLFOA could allow detection of HIV-1 infections that are missed by the conventional ELISA at the window period during acute infection to further reduce the risk for HIV-1 infection due to the undetected HIV-1 in the blood products. Moreover, NLFOA can be easily applied to more sensitive detection of other antigens.

## Introduction

Enzyme-linked immunosorbent assay (ELISA) is a widely used method for detection of proteins such as hormones, immunoglobulins and infectious agent antigens because of its sensitivity and easiness to use [1–3]. A typical sandwich ELISA method has been widely used for antigen detection to date. Fluorescent substrate (4-methylumbelliferyl phosphate, 4-MUP) was thought to be more sensitive than colorigenic substrate and has been explored to increase the sensitivity of ELISA [4]. Although a 2 or 100-fold increase in sensitivity was reported [4,5], the improvement was not consistently reproduced in other studies [6–8]. Recently, the unique properties of nanoparticles (large ratio of surface area-to-volume, stability and high binding capability) were explored to improve ELISA sensitivity [9–12]. Because of their high sensitivity and specificity, many nanoparticles have been generated for diagnosis of diseases [13,14]. In all previous ELISA systems, the enzymes conjugated to the detection antibodies are horseradish peroxidase (HRP), alkaline phosphatase (ALP), β-D-galactosidase, glucose oxidase or malate dehydrogenase [15–18]. They share some common features: stable, highly active, and capable of forming cross-links with other proteins (antibody, streptavidin and others). To date, no other enzymes have been used to further improve the ELISA sensitivity.

The nucleocapsid protein p24 of HIV-1 Gag has been widely used as a surrogate marker for HIV-1 infection [19–22]. A large number of viral particles are produced after infection during acute infection stage [23–25]. Thus, the detection of p24 and HIV-1 specific antibodies has been used for diagnosis of HIV-1 infection. This has prevented the vast majority of new HIV-1 infections due to the transfusion of HIV-1 positive blood [19,26]. Generally, the p24 concentration is too low between infection and the first time when it is detectable (window period) by the conventional ELISA [27,28]. Thus, individuals who are infected with HIV-1 during this window cannot be diagnosed by ELISA. The third and fourth-generation ELISA, which use denatured plasma samples, have improved the detection of early HIV-1 infection [29–31]. However, this window period was not significantly shortened while the complexity of testing was increased [32]. The ultrasensitive immunoassay (immune complex transfer enzyme immunoassay) was more sensitive for detection of p24 (> 630-fold), but this procedure was tedious and the serious serum interference limited its application [33]. Recently, nanoparticle based assays were reported to be able to significantly lower the detection limit of p24 than the conventional ELISA [34–36]. However, the complex procedures, high cost, the need for rare metal and the inherently inaccurate quantification of target molecules limited their wide use. Therefore, the sophisticated and expensive reverse transcription-polymerase chain reaction (RT-PCR) method was still the only assay capable of efficiently detecting early HIV-1 infection at the window period [26].

To develop a sensitive method for detection of low concentrations of the HIV-1 p24 antigen, we established a novel nuclease-linked fluorescence oligonucleotide assay (NLFOA) that coupled the advantages of nanoparticle technology, the nuclease and the fluorescent labeled oligonucleotides (FLOS) as the substrate. The new NLFOA method is 10-fold more sensitive than the conventional ELISA. NLFOA is also highly specific, reproducible and user-friendly. Since NLFOA can be easily applied to detection of any antigens, it can be used as a powerful tool to detect low concentration antigens, shorten the window period of infectious diseases, and diagnose other diseases.

## Materials And Methods

### Design of fluorescent labeled oligonucleotide substrate (FLOS)

The oligo BAM (5’-Iowa Black FQ/GCGGATCCCGCCTCTTGAGGCGGGATCCGC/Joe -3’) and the oligo ECO (5’-Iowa Black FQ/GCGAATTCCGCCTCTTGAGGCGGAATTCGC/Joe -3’) were synthesized by Integrated DNA Technology (Coralville, IA, U.S.A). The sites recognized by the restriction enzymes BamH I and EcoR I were indicated by underline. Both oligos were free of secondary structures and not homologous to sequences in the GenBank as determined by BLAST searching. Since they were labeled with a quencher Iowa Black FQ at the 5’ end and a fluorophore Joe at the 3’ end, no fluorescence could be detected from the oligo itself. The oligo could form a perfect dimer after the oligos were heated at 94°C for 5 minutes and annealed at 25°C for 2 minutes. After the oligodimer was digested by restriction enzymes BamH I or EcoR I, or after single- or double-stranded oligos were digested by exonuclease, the fluorophore Joe was released for detection.

### Determination of enzyme relative activity

To identify a nuclease with the high relative activity, we tested nine nucleases: TurboNuclease (Accelagen, San Diego, CA, U.S.A.); Mung Bean Nuclease, S1 Nuclease, BAL 31 Nuclease, Exonuclease III, Exonuclease I as well as BamH I and EcoR I restrictive endonucleases (Takara, Dalian, Liaoning, China); and DNase I (Thermo Fisher Scientific, Waltham, MA, U.S.A.). The purity and concentration of each enzyme was determined on a sodium dodecylsulfate polyacrylamide gel (SDS-PAGE). Each commercial nuclease was mixed with the equal volume of 2× sample buffer (containing 0.5 M Tris-HCl pH 6.8, 25%; 0.27 M 2-Mercaptoethanol; SDS, 0.14 M; bromophenol blue, 0.003 M; glycerol, 20%; H_2_O, 50%), and the mixtures were then boiled for 2 minutes. Each sample (10 μL) was loaded on the gel and electrophoresed for 1.5 hours at 120 V on a Mini Protean Tetra Cell (BIO-RAD, Hercules, CA, U.S.A.). The gel was stained with Coomassie blue and scanned on an odyssey CLx infrared imaging system (LI-COR, Lincoln, NE, U.S.A.) at the 700 nm. The signal intension of each band on SDS-PAGE was quantified by the software of Image Studio Ver. 2.1 (LI-COR, Lincoln, NE, U.S.A.).

For determination of enzyme relative activity, each enzyme was diluted at 10-fold serial dilutions in the enzyme specific buffer then incubated with the FLOS substrate at 37°C for 100 minutes. The fluorescent signals were measured on a Chromo 4 System (Bio-Rad, Hercules, CA, U.S.A.) to determine the enzyme activity kinetics by reading the plate every 5 minutes. The data was analyzed by the software Opticon Monitor 3 (Bio-Rad, Hercules, CA, U.S.A.). The enzyme relative activity was calculated by dividing MFI with absolute weight of the enzyme (MFI/ng) for each enzyme. The experiment was repeated three times.

### Nuclease-linked fluorescence oligonucleotide assay (NLFOA)

**Fig 1:**
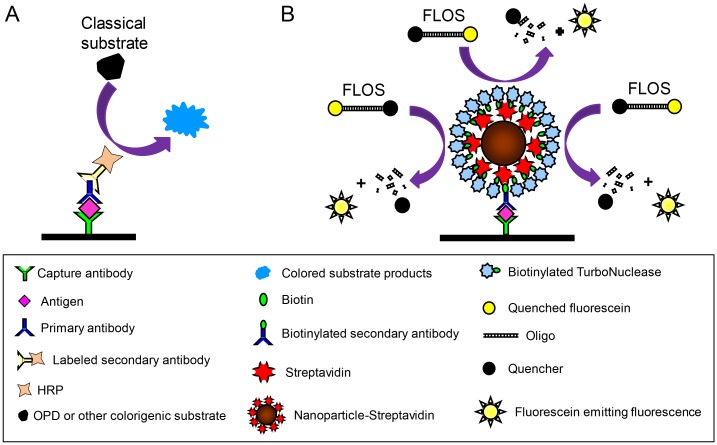
Schematic presentation of the nuclease-linked fluorescence oligonucleotide assay (NLFOA). (A) In the conventional ELISA, the capture antibody-antigen complex binds to one primary antibody and subsequently to one enzyme-conjugated second antibody. The enzyme acts on the substrate to generate colored substrate products. The reaction is detected by measuring absorbance at a certain wavelength of the chemical reaction. (B) In NLFOA, the capture antibody-antigen complex binds to one biotinylated primary antibody, then to the nanoparticle-streptavidin complex containing multiple copies of streptavidins, and finally to multiple copies of biotinylated TurboNuclease. The fluorescent labeled oliogonucleotide substrate (FLOS), which does not emit fluorescence due to the Iowa Black FQ (quencher) at the other end of the oligo, can generate fluorescence after digestion by TurboNuclease.

The NLFOA procedure was similar to that in the classical HIV-1 p24 ELISA (PerkinElmer, Waltham, MA, U.S.A.), except that Streptavidin (SA)-HRP and OPD (o-phenylenediamine dihydrochloride) were replaced with nanoparticle-streptavidin (Innova Biosciences, Cambridge, Cambs, U.K.), biotinylated TurboNuclease (bio-TurboNuclease) and the FLOS substrate (Fig 1). Bio-TurboNuclease was prepared using the ImmunoProbe Biotinylation Kit (Sigma Aldrich, Saint Louis, MO, U.S.A.). The 96-cell polystyrene microplate was coated with the p24 monoclonal antibody (from PerkinElmer HIV-1 p24 ELISA kit) and blocked with blocking buffer (PBS containing 5.0 g/L defatted milk powder). After p24 was added and the plate was incubated at 37°C for two hours, the biotinylated anti-p24 antibody (from PerkinElmer HIV-1 p24 ELISA kit) was added and incubated at 37°C for one hour. Nanoparticle-streptavidin was then added and incubated at 37°C for 40 minutes. After bio-TurboNuclease was added and incubated at 37°C for 40 minutes, FLOS in TurboNuclease reaction buffer (50 mM Tris-HCl, pH 8.0 and 1 mM MgCl_2_) was added and incubated at 37°C for 40 minutes. The fluorescence intensity was measured on a VICTOR X2 Series Multilabel Plate Reader (PerkinElmer, Waltham, MA, U.S.A.). A washing step with PBS containing 0.2% Tween 20 was performed between each step.

### Determination of the sensitivity, specificity and reproducibility of NLFOA

The10-fold serial dilutions of p24 in the dilution buffer (PBS containing 1 g/L bovine serum albumin) and spiked in a serum from an HIV-1 negative individual were detected by NLFOA and the conventional ELISA (HIV-1 p24 ELISA). One hundred sera from HIV-1 negative individuals were included as negative controls. The written consent was obtained from all subjects who participated in the study, and the study was approved by the ethics committee of Jilin University. HIV-1 p24 was diluted at low (1 pg/mL), intermediate (10 pg/mL) and high (100 pg/mL) concentrations for determination of the reproducibility and the coefficient of variation (CV). To compare the sensitivity and reproducibility between NLFOA and ELISA, the intra-assay variation was determined with p24 spiked samples (*N* = 5) analyzed on the same day, and the inter-assay variation was determined with the same samples analyzed on different days. All comparisons were carried out in five independent repeats.

To demonstrate the applicability of NLFOA for detection of other antigens, the capsid protein VP1 of enterovirus 71 (EV71) was analyzed. The procedure for detection of VP1 was similar to that used for p24 detection, with the following changes: mAb 2H2 targeting VP1 was used to coat 96-cell polystyrene microplate, recombinant VP1 expressed in *E*. *coli* was spiked in sera, and the biotinylated detection antibody was prepared from VP1-immunized rabbits.

### Comparison of the signal/noise (S/N) ratio between ELISA and NLFOA

As the indication of the assay precision (the variation coefficient in signal intensity value), S/N ratios of ELISA and NLFOA were determined with results from three independent replicates at various p24 concentrations spiked in sera. The S/N ratio was calculated using the total standard deviation (here denoted *σ*_total_, and the Eq is that

$$\sigma_{\text{total}}=\sqrt{\sigma_{s}^{2}+\sigma_{c}^{2}}$$

in which the *σ*_*s*_ is the SD of the sample and *σ*_*c*_ is the SD of the negative control), the average signal of the sample (*μ*_*s*_) and the average signal of the negative control (*μ*_*c*_). S/N values were determined according to the Eq *S/N* = (*μ*_*s*_ - *μ*_*c*_)/*σ*_total_. [4]

### Statistical analysis

Statistical analysis was performed using IBM SPSS Statistics 21 (IBM, Armonk, NY, U.S.A.) and Microsoft Excel software (Microsoft Corp., Redmond, WA, U.S.A.). Differences in mean values were analyzed by student’s *t* test. *P* values less than 0.05 were considered significant.

## Results

### Identification of nuclease with the highest relative activity

**Fig 2:**
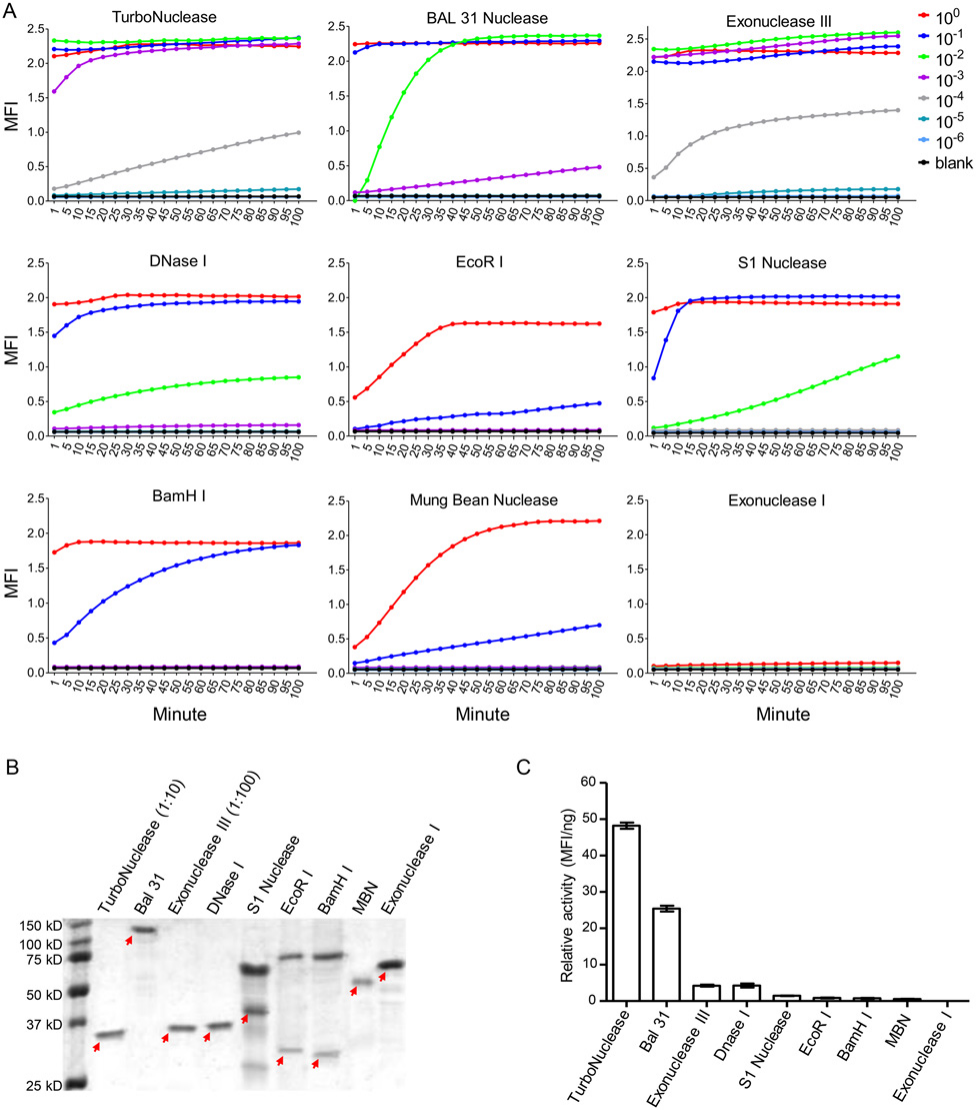
Determination of relative activity of different nucleases. (A) The nuclease activity was determined by mixing FLOS with serially diluted enzymes (1:10). The mean fluorescence intensity (MFI) was measured every 5 minutes. (B) Analysis of the enzyme contents in the commercial enzyme stocks by SDS-PAGE. Equal volume (10 μl) of each commercial nuclease was loaded onto a SDS-PAGE gel for electrophoresis. Exonuclease III and TurboNuclese were diluted at 1:100 and 1:10, respectively, due to the high protein concentration in the samples. (C) Determination of enzyme relative activity. The relative activity of each enzyme was defined by the positive MFI (2.1 folds above the background) at the lowest concentration of the enzyme (MFI/ng). All experiments were independently performed three times and results from one representative experiment are shown. Negative control (NC) contained all components except the enzyme. Means ± SD are shown.

To identify a nuclease with high relative activity as the coupling enzyme, we tested a total of nine enzymes: seven nucleases (TurboNuclease, Mung Bean, S1, BAL 31, Exonuclease III, Exonuclease I and DNase I) and two restrictive endonuclease (EcoR І and BamH I). Each enzyme was diluted at 1:10 serial dilutions and mixed with the fluorescent labeled oligonucleotide substrate (FLOS) (10^–6^ M) to release the fluorophore that was quenched by Iowa Black FQ. The kinetic of the mean fluorescence intensity (MFI) was measured every five minutes. The positive fluorescent signal could be detected for both TurboNclease and Exonuclease III at the 1:10,000 dilution, BAL 31 at the 1:1,000 dilution, DNase I and S1 at the 1:100 dilution, and BamH I, EcoR I and Mung Bean at the 1:10 dilution (Fig 2A). Only very weak signal was detected for Exonuclease I when it was not diluted. These results showed that TurboNuclease and exonuclease III had the highest activity.

To determine the relative activity of each enzyme, we first determined the proportion of the enzyme protein in the stock by SDS-PAGE. One predominant enzyme protein was detected for TurboNuclease, Bal 31, Exonuclease III, DNase I, Mung Bean and Exonuclease I, while other proteins beside the enzymes were also present for S1 nuclease, EcoR I and BamH I (Fig 2B). When only the enzyme proteins were compared, similar concentrations (40–80 μg/mL) were observed for Bal 31, DNase I, S1 nuclease, EcoR I, BamH I, Mung Bean and Exonuclease I. The concentrations of TurboNuclease and Exonuclease III were about 10- and 100-fold higher than those of other enzymes, respectively. We then determined the relative activity for each enzyme by dividing MFI with absolute weight of the enzyme (MFI/ng) in the stock. The relative activity was the highest for TurboNuclease (48 MFI/ng) and the second highest for BAL 31 (26 MFI/ng) (Fig 2C). All other enzymes had much lower relative activity. Two restrictive endonucleases EcoR I and BamH I were able to recognize the specific nucleotides in the double stranded FLOS substrates. However, the signals generated by either enzyme were too weak to be applicable. These results demonstrated that TurboNuclease had the highest relative activity and was used for all subsequent experiments.

### Determination of the minimal concentration of TurboNuclease required for NLFOA

**Fig 3:**
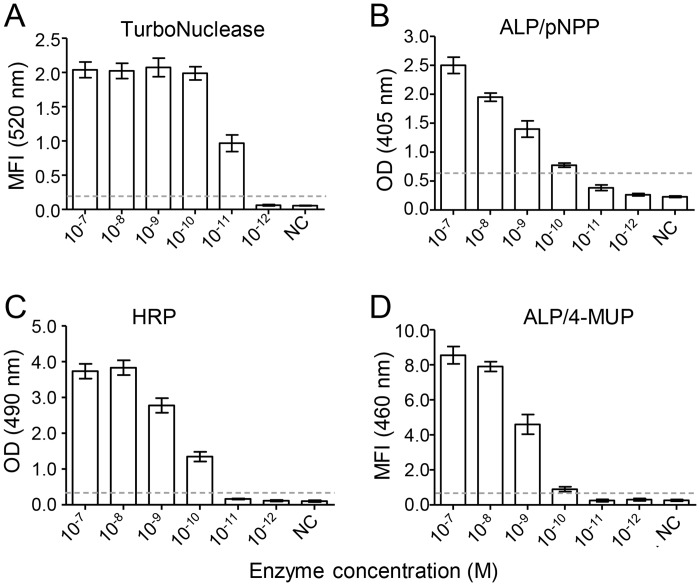
Comparison of minimal required enzyme concentrations among different detection systems. The lowest concentration of the enzyme required for detection was determined for four different enzyme-substrate systems: (A) TurboNuclease and FLOS system; (B) alkaline phosphatase (ALP) and p-nitrophenylphosphate (pNPP) system; (C) horseradish peroxidase (HRP) and o-Phenylenediamine (OPD) system; and (D) ALP and 4-methylumbelliferyl phosphate (4-MUP, a classical fluorogenic substrate) system. Each enzyme was prepared at 1:10 serial dilutions (starting at 10^–7^ M) and reacted with its corresponding substrate. Negative control (NC) contained all components except the enzyme. The cutoff (2.1 fold higher than the NC signal) is indicated by the dotted line. Means ± SD are shown.

We next determined whether the enzyme concentration required for generation of detectable signals was the lowest in the TurboNuclease-FLOS system by comparing it to three other commonly used enzyme-substrate system (HRP-OPD, ALP-pNPP and ALP-4-MUP). In each system, the enzyme was diluted at 1:10 serial dilutions, starting at 10^–7^ M and reacted with the optimal concentration of corresponding substrates. A positive signal was detected at 10^–11^ M for TurboNuclease (Fig 3A), while it was at 10^–10^ M for the enzyme-colorigenic substrate (pNPP or OPD) system (Fig 3B and 3C) and ALP-4MUP fluorogenic substrate system (Fig 3D). Thus, 10-fold less of TurboNuclease than other enzymes was needed to generate detectable signals. Some studies showed that fluorescent substrate 4-MUP was able to improve the ELISA sensitivity [5,7,37], but no improvement was reported in other studies [6–8]. Our results also did not show any improvement in sensitivity with fluorescent substrate 4-MUP (Fig 3D). These data demonstrated that the novel NLFOA method that used both the TurboNuclease and FLOS could be developed as a highly sensitive assay.

**Fig 4:**
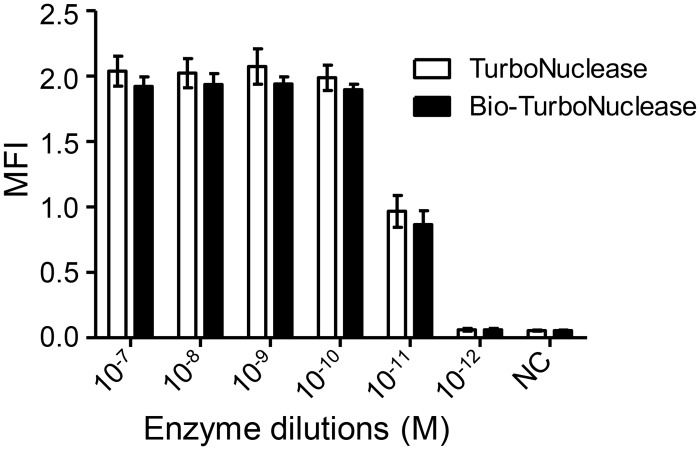
Similar enzyme activity between TurboNuclease and bio-TurboNuclease. The mean fluorescence intensity (MFI) was compared between TurboNuclease and bio-TurboNuclease at 1:10 serial dilutions (p>0.2, Student’s *t* test). Each assay was performed in five independent experiments. Means ± SD are shown.

Since the Turbonuclease needed to be biotinylated in NLFOA, we next determined whether biotinylation could affect the enzyme activity. When compared to the unmodified TurboNuclease, bio-TurboNuclease had similar activity (p = 0.344, Student’s *t* test), suggesting that biotinylation of TurboNuclease had no or minimal impact on enzyme activity (Fig 4).

### Optimization of NLFOA

**Fig 5:**
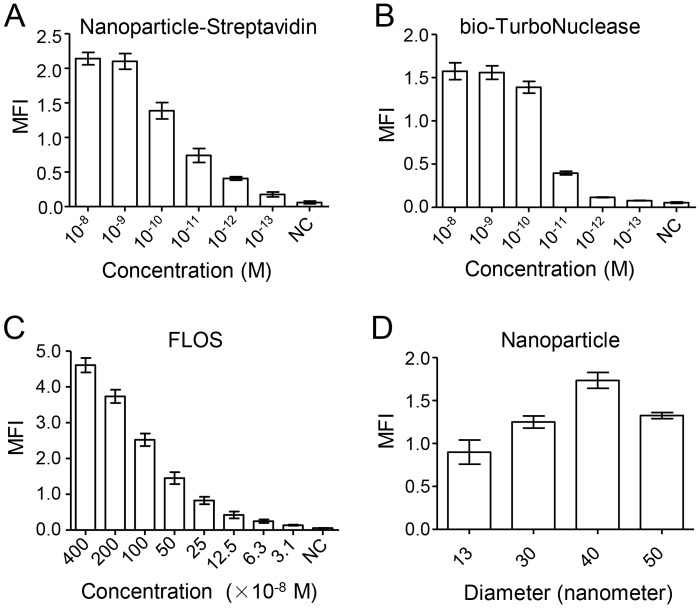
Optimization of the components in NLFOA. The NLFOA conditions were optimized for (A) nanoparticle-streptavidin, (B) bio-TurboNuclease, (C) FLOS and (D) diameters of the nanoparticles. Both nanoparticle-streptavidin and bio-TurboNuclease were prepared at 1:10 serial dilutions between 10^–8^ -10^-13^ M. The FLOS oligos were prepared at 1:2 serial dilutions between 400–3.1 ×10^–8^ M. Four different diameter sizes (13, 30, 40 and 50 nm) were analyzed for nanoparticles. Negative control (NC) contained all components except what was to be optimized. Each of the component was analyzed in five repeats and were performed in two independent experiments. Means ± SD are shown.

To optimize the conditions for NLFOA, we tested various concentrations of nanoparticle-streptavidin, bio-TurboNuclease and FLOS as well as the diameters of nanoparticles. When one component was optimized for NLFOA, all other components were kept at excessive concentrations. MFI for nanoparticle-streptavidin was similar at 10^–8^ M and 10^–9^ M, reduced at 10^–10^ M, but significantly decreased at lower concentrations (10^-11^-10^-13^ M) (Fig 5A). MFI was similar at concentrations 10^–8^, 10^–9^, and 10^–10^ M for bio-TurboNuclease, but significantly decreased at lower concentrations (10^-11^-10^-13^ M) (Fig 5B). To determine how the FLOS concentrations affected assay system, we made 1:2 serial dilutions from the 4 × 10^–6^ M stock. The MFI was the highest at the 400 × 10^–8^ M of FLOS. It was continuously reduced as the FLOS concentrations decreased. The signal at 100 × 10^–8^ M was less than two-fold lower than that for the highest stock (400 × 10^–8^ M), but it was still significantly higher than the background (Fig 5C). However, the FLOS concentration at 100 × 10^–8^ M was more cost effective. Finally, we determined the influence of the size of the nanoparticle spheres on the assay signals. Among four different sizes tested, the nanoparticles with diameter of 40 nm yielded the strongest signal (Fig 5D). Taken together, these results showed that nanoparticles with a diameter of 40 nm, nanoparticle-streptavidin at 10^–9^ M, bio-TurboNuclease at 10^–10^ M, and FLOS at 10^–6^ M were optimal for NLFOA. These conditions were used for all the rest of the experiments.

### The enhanced signal by nanoparticle-streptavidin and FLOS

**Fig 6:**
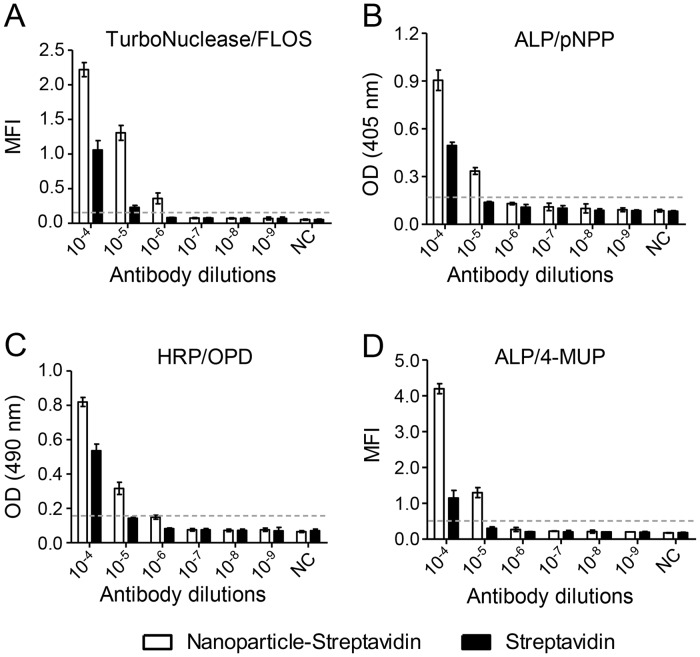
Increased sensitivity with nanoparticle-streptavidin and FLOS. The signals generated by the nanoparticle-streptavidin and streptavidin systems were compared among four different enzyme-substrate systems: (A) TurboNuclease and FLOS; (B) alkaline phosphatase (ALP) and p-nitrophenylphosphate (pNPP); (C) horseradish peroxidase (HRP) and o-Phenylenediamine (OPD); and (D) alkaline phosphatase (ALP) and 4-methylumbelliferyl phosphate (4-MUP). Each enzyme was prepared at 1:10 serial dilutions (10^–4^–10^–9^ M). Negative control (NC) contained all components except the enzyme. The cutoff (2.1 fold higher than the NC signal) is indicated by the dotted line. Means ± SD are shown.

To investigate whether the nanoparticle and FLOS could generate a more enhanced signal in NLFOA than other enzyme-substrate systems, we replaced streptavidin with nanoparticle-streptavidin in NLFOA and three other ELISA systems by testing 1:10 serial antibody dilutions. The nanoparticle-streptavidin increased the sensitivity by 10-fold, compared to the streptavidin alone (Fig 6A). Moreover, the FLOS substrate detected the antibody dilution at 1:10^–5^, while the OPD and pNPP colorigenic substrates (Fig 6B and 6C) and the 4-MUP fluorogenic substrate (Fig 6D) only detected the antibody dilution at 1:10^–4^. These results showed that the novel NLFOA method, which incorporated the FLOS substrate, nuclease and nanoparticle, was more sensitive than three conventional ELISA systems.

### Determination of the detection limit of NLFOA

**Fig 7:**
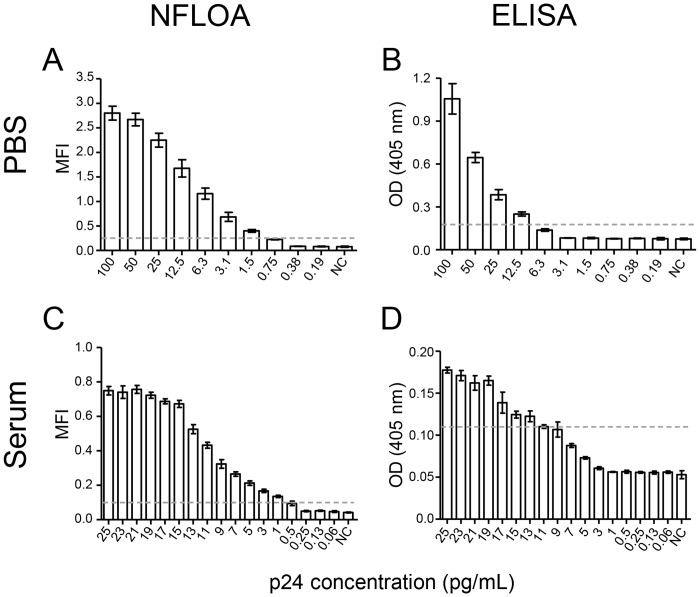
Comparison of the detection limit between NLFOA and the conventional ELISA. The detection limit for HIV-1 p24 antigen diluted in PBS by NLFOA (A) and conventional ELISA (B). The detection limit of HIV-1 p24 antigen spiked in sera from a healthy human by NLFOA (C) and conventional ELISA (D). The dotted line indicates the cutoff value that is 2.1-fold of that of the negative control (NC). Experiments with p24 in PBS (A and B) were done in five repeats and experiments with p24 in sera (C and D) were done in eight repeats. Each assay was performed in three independent experiments. Means ± SD are shown.

To investigate whether NLFOA could be used as a more sensitive method for detection of p24, we next determined its detection limit in samples. First, p24 was prepared at 1:2 serial dilutions in PBS and analyzed in parallel by NLFOA and the commonly-used p24 detection ELISA kit (PerkinElmer, Waltham, MA, U.S.A.). NLFOA detected p24 at a concentration of 1.5 pg/mL (Fig 7A), while ELISA could only detect p24 at a concentration of 12.5 pg/mL (Fig 7B). Thus, NLFOA was about 10-fold more sensitive than ELISA. To investigate whether NLFOA could detect p24 in human sera at the similar sensitivity level, we spiked the p24 in a serum from an HIV-1 negative individual at concentrations from 0.25–25 pg/mL. Since both NLFOA and ELISA could detect p24 at concentrations >25 pg/ml, higher p24 concentrations were not analyzed. The concentration of p24 as low as 1 pg/mL could be consistently detected by NLFOA (Fig 7C), while only the p24 concentrations at 11 pg/mL or higher could be consistently detected by ELISA (Fig 7D). Further analysis of intra- and inter-assay variation showed that the detection limit of NLFOA (0.9 pg/mL) was significantly lower than that of ELISA (10.8 pg/mL) (p<0.0001; Table 1). These results demonstrated that NLFOA was about 10-fold more sensitive than ELISA for detection of HIV-1 p24 present in blood samples.

**Table 1:** Comparison of sensitivity and reproducibility between NLFOA and ELISA.

Assay	Sensitivity (pg/mL)			Reproducibility (% of CV)		
	NLFOA	ELSA	P	NLFOA	ELSA	P
Intra-assay	0.9 ± 0.2	11 ± 1.4	<0.0001	7.3 ± 0.7	8.6 ± 0.8	0.03
Inter-assay	0.8 ± 0.3	10.6 ± 1.7	<0.0001	7.1 ± 0.4	8.7 ± 0.8	0.01
Overall	0.9 ± 0.2	10.8 ± 1.5	<0.0001	7.2 ± 0.6	8.6 ± 0.8	<0.0001

The values are shown as mean ± SD of the data from five independent experiments.

**Fig 8:**
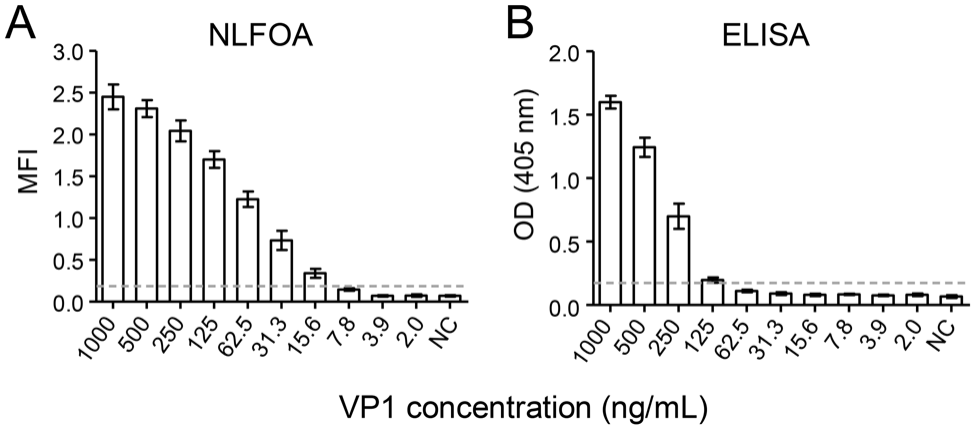
Detection of the EV71 VP1 antigen by NLFOA. The EV71 VP1 antigen was spiked in sera and anzlysed in parallel by NLFOA (A) and the conventional ELISA (B). The dotted line indicates the cutoff value that is 2.1-fold of that of the negative control (NC). Each assay was performed in three independent experiments. Means ± SD are shown.

To investigate if NLFOA could be adapted to detection of other antigens, we used NLFOA to detect the capsid protein VP1 of enterovirus 71 (EV71). The recombinant VP1 was prepared at 1:2 serial dilutions in sera and then analyzed in parallel by NLFOA and conventional ELISA. VP1 at 15.6 ng/mL could be consistently detected by NLFOA, while VP1 at 7.8 ng/mL was occasionally detected (Fig 8A). In contrast, VP1 at 125 ng/mL was consistently detected by the conventional ELISA method, while VP1 at the next dilution (62.5 ng/mL) could not be detected (Fig 8B). These results demonstrated that NLFOA was also ~10-fold more sensitive for detection of the VP1 antigen than ELISA.

### Specificity and reproducibility of NLFOA

We next determined the specificity of NLFOA using two panels of samples: 100 sera from HIV-1 negative individuals and 170 serum samples spiked with p24 at 0.5–60 pg/mL (Table 2). None of the 100 HIV-1 negative samples were positive by NLFOA or ELISA. NLFOA could detect p24 in all spiked serum samples at ≥ 1 pg/mL and 50% of the samples at 0.5 pg/mL. In contrast, ELISA could only detect p24 in the spiked serum samples at ≥ 15 pg/mL and 92% of the samples at 10 pg/mL, but could not detect p24 in samples ≤ 6 pg/mL. These results demonstrated that NLFOA was as specific as the conventional ELISA, but more sensitive.

**Table 2:** Sensitivity and Specificity of the NLFOA method.

p24 concentration (pg/mL)	No. of repeats	NLFOA	ELISA
		% of positive repeats	% of positive repeats
0.5	20	50	ND
1	20	100	ND
2	25	100	ND
4	15	100	ND
6	15	100	ND
10	25	100	92
15	20	100	100
30	15	100	100
60	15	100	100
Negative control	100	0	0

ND: not detected

The reproducibility of NLFOA was evaluated with three panels of the serum samples spiked with p24: low concentration (1 pg/mL), intermediate concentration (10 pg/mL), and high concentration (100 pg/mL). Twenty repeats were used for the low concentration samples, while 15 repeats were used for intermediate and high concentration samples. The coefficient of variation (CV) was determined to evaluate the assay reproducibility for each panel. The CVs were 7.8%, 9.1% and 8.4% for the low, intermediate and high concentration panels, respectively; all were lower than the accepted level (10%). To compare the reproducibility between NLFOA and ELISA, low p24 concentration samples were analyzed by both methods. The CV of NLFOA was significantly lower than that of ELISA: 7.3 vs. 8.6 for intra-assay (p = 0.03) and 7.1 vs. 8.7 for inter-assay (p = 0.01) (Table 1). Overall, the reproducibility of NLFOA was significantly better than that of ELISA (p<0.001). These results showed that NLFOA was a more reproducible than ELISA.

### The signal/noise (S/N) ratio of the NLFOA

**Fig 9:**
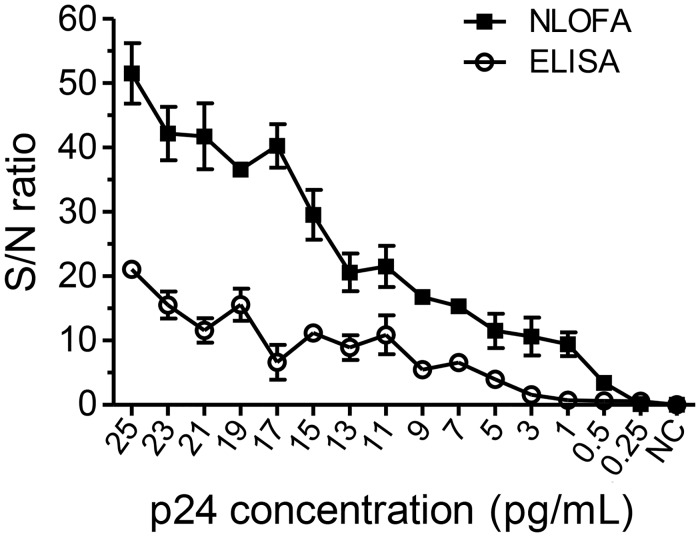
Enhanced precision with NLFOA. The signal/noise (S/N) ratios were determined for both NLFOA and ELISA assays at different p24 concentrations.

The S/N ratio of NLFOA was determined with various p24 concentrations and compared to that of ELISA. The S/N ratio values of NLFOA had a steeper upward trend than those of ELISA as the analyte concentration increased. The NLFOA S/N ratios were significantly (p = 0.014) higher (2.4 to 6.2-fold) than those of ELISA at concentrations at which both assays yielded the positive results (Fig 9).

## Discussion

ELISA is one of the most commonly used methods to detect antigens using the enzymes as surrogates because of its specificity, sensitivity, consistency, easiness of use and low cost. However, the need to detect low concentrations of antigens in samples in clinical diagnosis and laboratory research demands development of more sensitive assays. In the present study, we developed a novel nuclease-linked fluorescence oligonucleotide assay (NLFOA) by taking the advantages of the nanoparticles coupled with multiple streptavidin copies, high affinity and specificity of streptavidin-biotin interaction, biotinylated TurboNuclease and the novel fluorescent labeled oligonucleotide substrate (FLOS). NLFOA was 10-fold more sensitive than the conventional ELISA assay. Moreover, NLFOA is highly specific, reproducible, low cost and user-friendly.

The increased sensitivity of NLFOA could be contributed by two improvements: the signal amplification by nanoparticles coupled with the nuclease and a novel fluorescent labeled oligonucleotides (FLOS) substrate. A nanoparticle could be coupled with many streptavidin molecules that could bind to additional multiple bio-TurboNucleases (Fig 1B). This would result in more substrate becoming catalyzed for amplification of detection signals as previously reported [10,12,38,39]. Previously, fluorescent substrate 4-MUP was found to be able to improve the ELISA sensitivity [4,5], but no improvement was reported in other studies [6–8]. We also tested the 4-MUP substrate system but did not observe any improvement in assay sensitivity (Figs 3D and 6D), similar to the results reported by others [6–8]. Instead, we developed a novel fluorescent labeled oligonucleotide substrate (FLOS). The fluorophore Joe at the 3’ end was fully quenched the by the quencher Iowa Black FQ at 5’ end in the same FLOS oligo. The fluorescent could only be released for detection after the FLOS was digested by nuclease. Thus, the fluorescent signal was intense and had a high signal/noise ratio [40,41], which was critical for high sensitivity, specificity and reproducibility of NLFOA. Because S/N ratio is an important factor of the assay precision including sensitivity, reproducibility and specificity, the better S/N ratio of NLFOA suggested that NLFOA could more reliably detected antigens present at very low concentrations than the conventional ELISA. By analyzing a large number of p24 negative and positive samples, we also demonstrated that NLFOA was as specific and reproducible as the previously well-established ELISA assays [42,43].

Since detection of p24 by a highly sensitive ELISA-based method can play a critical role in diagnosis of new HIV-1 infections, many sensitive p24 detection assays have been developed [33–36]. However, the complex procedures, high cost, and the inherently inaccurate quantification of target molecules become major hurdles for their wide use. Recently, two highly sensitive nanoparticle-based bio-barcode amplification assays were developed for detection of HIV-1 p24 antigen present as low as 0.1 pg/mL [36]. The new assays combined both the immunoassay and the PCR method together. The p24 concentration was determined by PCR amplifying the oligos attached to the nanoparticles that were captured though anti-p24 antibodies using gel electrophoresis. The assays were based on an ELISA system that could only detected p24 at 1,000 pg/mL. If higher affinity p24 mAbs as in the commercial p24 ELISA kit were used in both assays, the p24 detection limit could be even lower. However, since p24 was tested only in PBS with BSA for both assays in the report, it is not clear how plasma samples will affect the assays’ sensitivity and specificity. In addition, PCR was required in both systems. It is not clear if both assays will be as sensitive as the single PCR assay that detect viral RNA copies in virions since the head-on comparison was not reported. The PCR cost, cumbersome gel electrophoresis and extra steps make both nanoparticle-based bio-barcode amplification assays unlike to be widely used for analyzing large numbers of samples. In contrast, NLFOA increased the sensitivity of ELISA by using nuclease, FLOS oligo and nanoparticles, but without the additional PCR step. Although NLFOA did not increase the sensitivity for p24 detection as high as these two nanoparticle-based bio-barcode amplification assays, the combination of the sensitivity, specificity, reproducibility, low cost and user-friendly procedure makes it easy to be adapted for detection of p24 or any other antigens.

A number of nucleases were tested to establish the NLFOA method. Two restrictive endonucleases EcoR І and BamH І were tested for their ability to recognize the specific nucleotides in the double stranded FLOS oligos. However, signals generated by either enzyme were too low to be useful. Among seven tested nucleases, TurboNuclease yielded the highest relative activity. TurboNuclease is an extracellular endonuclease from *Serratia macescens*. It nonspecifically hydrolyzes both single- and double-stranded DNA and RNA to small 5’-phosphorylated oligonucleotides (1–4 bases). We for the first time demonstrated that TurboNuclease together with novel fluorescent labeled oligonucleotide substrates could be used improve the sensitivity of the ELISA-based assays.

There were three stages that could amplify the initial antigen-antibody interaction signal (Fig 1). First, the single biotinylated antibody bounded to one nanoparticle could be coupled with many streptavidin molecules (a maximum of ~ 256 streptavidin molecules) [44]. Second, one streptavidin could bind to up to three bio-TurboNucleases. Third, a number of FLOS molecules could be digested by bio-TurboNuclease. After three stages of amplification of the antigen-antibody interaction signal, a much higher sensitivity was expected in NLFOA. However, only about a 10-fold increase in sensitivity in NLFOA compared to the conventional ELISA was observed in this study. This suggested that a better understanding of the limiting steps could lead to further improvement in sensitivity of NLFOA.

**Fig 10:**
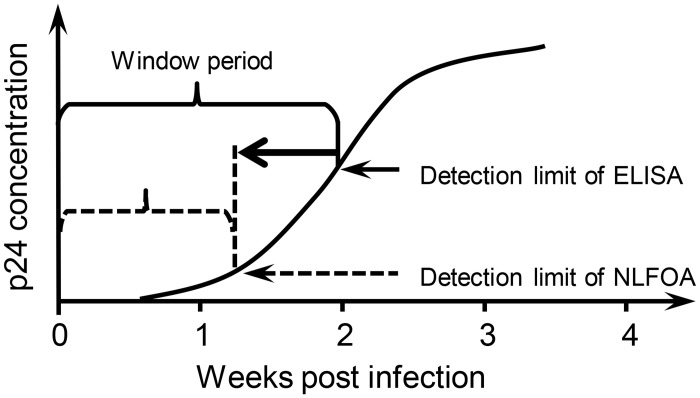
Shortened window period of HIV-1 infection by NLFOA. The HIV-1 p24 antigen can only be detected after a period of time after HIV-1 infection (window period) by the conventional ELISA. The higher sensitivity of NLFOA can likely shorten this window period.

The improved sensitivity of NLFOA can have a significant impact on the clinical diagnosis of HIV-1 and other pathogens. For example, the p24 antigen, which is highly cross-reactive among HIV-1 group M subtypes [45], was the first HIV-1 antigen that can be detected [24,26]. However, the window period from infection to the first time when p24 can be detected is about 14 days (Fig 10) [19,24,25,46,47]. If donated blood is collected during this period, the HIV-1 infection cannot be diagnosed by the conventional ELISA even though the blood at this time can be highly contagious. The viral RNA can be detected at this time by the RT-PCR assay, but it can not be routinely employed in resource limited regions due to high costs and sophistication of procedure [48]. Since NLFOA is about 10 fold more sensitive than conventional ELISA, the use of NLFOA to screen donated bloods can likely shorten this window period and reduce the incidence of HIV-1 infection due to the transfusion of HIV-1 positive blood (Fig 10). NLFOA, like ELISA, is more cost effective and user-friendly than RT-PCR when individual samples are analyzed. It costs 20 times less than RT-PCR. When the pooled nucleic acid amplification testing (PNAAT) was used with pooled 10–100 samples, the cost will be reduced by ~10–100 folds, but with a reduced sensitivity for samples with low viral loads. However, positive pools need to be retested with subpools or individual samples. These will require additional time and tests to identify individual HIV-1 positive samples. Recent studies showed that PNAAT may be cost effective only in very high incidence settings, and more frequently testing with third- or fourth-generation immunoassay can be more economically efficient and may obviate the benefits of PNAAT for screening acute HIV-1 infections [49–51]. More sensitive and user-friendly immunoassays like NLFOA that can identify more acute HIV-1 infection cases in the eclipse window period may further increase the cost-effectiveness of immunoassays. More importantly, NLFOA can be easily adapted to detection of low levels of any proteins, as menstruated with the detection of EV71 VP1, and serves as a powerful tool for early diagnosis of infections or other diseases.
